# Disruption of the Cytoplasmic Membrane Structure and Barrier Function Underlies the Potent Antiseptic Activity of Octenidine in Gram-Positive Bacteria

**DOI:** 10.1128/aem.00180-22

**Published:** 2022-04-28

**Authors:** Nermina Malanovic, Jessica A. Buttress, Djenana Vejzovic, Ayse Ön, Paulina Piller, Dagmar Kolb, Karl Lohner, Henrik Strahl

**Affiliations:** a Institute of Molecular Biosciences, Biophysics Division, University of Graz, Graz, Austria; b BioTechMed Graz, Graz, Austria; c Centre for Bacterial Cell Biology, Biosciences Institute, Faculty of Medical Sciences, Newcastle University, Newcastle upon Tyne, United Kingdom; d Core Facility Ultrastructure Analysis, Center for Medical Research, Medical University of Graz, Graz, Austria; Centers for Disease Control and Prevention

**Keywords:** *Bacillus subtilis*, *Enterococcus hirae*, cell membrane, Gram-positive bacteria, lipid mutants, membrane activity, mode of action, octenidine

## Abstract

The antimicrobial killing mechanism of octenidine (OCT), a well-known antiseptic is poorly understood. We recently reported its interaction with Gram-negative bacteria by insertion of OCT into the outer and cytoplasmic membrane of Escherichia coli, resulting in a chaotic lipid rearrangement and rapid disruption of the cell envelope. Its action primarily disturbs the packing order of the hydrophobic moiety of a lipid, which consequently might result in a cascade of multiple effects at a cellular level. Here, we investigated OCT’s impact on two different Gram-positive bacteria, Enterococcus hirae and Bacillus subtilis, and their respective model membranes. In accordance with our previous results, OCT induced membrane disorder in all investigated model systems. Electron and fluorescence microscopy clearly demonstrated changes in cellular structure and membrane integrity. These changes were accompanied by neutralization of the surface charge in both *E. hirae and* B. subtilis and membrane disturbances associated with permeabilization. Similar permeabilization and disordering of the lipid bilayer was also observed in model membranes. Furthermore, experiments performed on strongly versus partly anionic membranes showed that the lipid disordering effect induced by OCT is a result of maximized hydrophobic over electrostatic forces without distinct neutralization of the surface charge or discrimination between the lipid head groups. Indeed, mutants lacking specific lipid head groups were also susceptible to OCT to a similar extent as the wild type. The observed unspecific mode of action of OCT underlines its broad antimicrobial profile and renders the development of bacterial resistance to this molecule less likely.

**IMPORTANCE** OCT is a well-established antiseptic molecule routinely used in a large field of clinical applications. Since the spread of antimicrobial resistance has restricted the use of antibiotics worldwide, topically applied antiseptics like OCT, with a broad spectrum of antimicrobial activity and high safety profile, gain increasing importance for effective infection prevention and therapy. To eliminate a wide spectrum of disease-causing microorganisms, a compound’s antiseptic activity should be unspecific or multitarget. Our results demonstrate an unspecific mechanism of action for OCT, which remained largely unknown for years. OCT disturbs the barrier function of a bacterial cell, a function that is absolutely fundamental for survival. Because OCT does not distinguish between lipids, the building blocks of bacterial membranes, its mode of action might be attributed to all bacteria, including (multi)drug-resistant isolates. Our results underpin OCT’s potent antiseptic activity for successful patient outcome.

## INTRODUCTION

The ongoing rapid emergence and global spread of (multi)drug antimicrobial resistance limit effective therapeutic options for successful patient outcome and constitute an enormous threat within health care facilities. In that regard, topically applied antiseptics represent a very useful tool for infection prevention and therapy. The synthetic antimicrobial molecule octenidine (OCT) has established itself over the last 30 years as a key agent for skin, mucous membrane, and wound antisepsis as well as microbial decolonization of patients (1–5) due to its potent activity against a wide range of multidrug-resistant Gram-negative and Gram-positive pathogens and fungi (6–9). OCT (*N*, *N′*-(1,10 decanediyldi-1[4H]-pyridinyl-4-ylidene) *bis*-(1-octanamine) dihydrochloride) is a quaternary ammonium compound of the bipyridine family exerting mesomeric distribution of the cationic charge via an amino-pyridine structure (10). The two cationic pyridine residues are separated by 10 methylene groups, and each amino-pyridine has a terminal hydrophobic octanyl group. Owing to this amphipathic character (hydrophobic and hydrophilic domains), OCT resembles membrane-active antimicrobial peptides (11), such as the well-described human antimicrobial peptides cathelicidin LL-37 (12) and lactoferricin (13). Its hydrophobic and cationic nature would allow for interaction with membrane components and, thus, efficient disruption of membranes. However, the detailed killing mechanism at a cellular and molecular level has remained largely unknown for years. In our previous work (10), we reported OCT’s interaction with Escherichia coli and model membranes mimicking Gram-negative bacteria. We used biophysical approaches to identify bacterial membrane components as the primary targets of OCT and proposed a sequential, rapid, and unspecific mode of action based on its physical interactions with bacterial phospholipids. OCT strongly compromises bacterial membrane integrity, while, on a molecular level, it induces a dramatic loss in the regular packing order of bacterial phospholipids. The degree of lipid disorder caused by OCT suggests the emergence of significant membrane ruptures and can be compared to compounds incorporating totally or partially in the hydrophobic core of the bilayer (14) or compounds inserting close to the polar/apolar interface (15–17).

In this study, we extend our observations to Gram-positive model organisms, in analogy to our previous work, to test if a similar membrane-active mode of action is also responsible for OCT’s effect against Gram-positive bacteria. Gram-positive bacteria contain a number of different components that may undergo interaction with OCT. Firstly, excluding some bacteria such as Mycobacteria and Corynebacteria, Gram-positive bacteria do not possess an outer membrane composed of phospholipids and lipopolysaccharides but a thicker peptidoglycan matrix in which negatively charged cell wall components, wall teichoic (WTA) and lipoteichoic (LTA) acids, are embedded. Furthermore, their single phospholipid membrane is characterized by the presence of exclusively anionic phosphatidylglycerol (PG) and cardiolipin (CL) (see review [18, 19]). Some Gram-positive species also exhibit higher levels of precursor molecules, such as phosphatidylserine (PS) and phosphatidic acid (PA) or glycolipids. Although some feature a considerable content of zwitterionic molecules, such as phosphatidylethanolamine (PE) or lysyl-phosphatidylglycerol (lysyl-PG), Gram-positive membranes are overall more negatively charged than those of their Gram-negative counterparts. Examples include Enterococcus hirae and Bacillus subtilis, which strongly differ in cell shape (diplococci versus classical rods) but have a very similar cell envelope architecture. Only the composition of their cytoplasmic membranes differs slightly, as *E. hirae* mainly contains the anionic phospholipids PG and CL (20); whereas B. subtilis is composed of both PG/CL and PE (21). Besides the phospholipids, a small quantity of glycolipids is present in the cytoplasmic membranes of both species. Thus, the composition of the B. subtilis cytoplasmic membrane is more similar to that of E. coli, differing primarily in the content of zwitterionic PE, which in B. subtilis occupies approximately 12% of the total phospholipid (PL) content versus 67% in E. coli. While the exact PL content may vary depending on growth phase, culture media, and the strain analyzed (18, 22, 23), B. subtilis membranes are generally found to be less anionic than their *E. hirae* counterparts. Such diversity in PL composition may influence OCT activity, as different physicochemical parameters of each PL determine the overall membrane properties. The common features of PL (18), besides being hydrophobic and diverse in their headgroups (neutral versus charged), include their capability to undergo hydrogen bonding. This allows the formation of different membrane shapes and curvatures, which may also impact their interactions with OCT.

Because of the differences in cell wall and phospholipid composition to Gram-negative bacteria, we examined the mode of action of OCT against Gram-positive bacteria using *E. hirae* and B. subtilis as model organisms, as well as corresponding model membranes mimicking the cytoplasmic membranes of those bacteria. The membrane architecture of both organisms (20, 21) is highly similar to the majority of Gram-positive bacteria, including pathogenic bacteria (24), and therefore highly suitable for investigating the mode of action of OCT in a relevant biological context. Whereas B. subtilis is one of the best characterized laboratory model strains, *E. hirae* is also associated with some pathogenicity in animals and rarely in humans (25).

## RESULTS AND DISCUSSION

### OCT rapidly kills *E. hirae* at low doses.

First, we estimated the antimicrobial activity of OCT for various inoculations of *E. hirae*, as each of our experiments required a different bacterial load. As shown in Table 1, MIC values of 1 mg/L OCT are lethal for 1 × 10^6^ CFU/mL *E. hirae*, which corresponds well to the published antimicrobial profile of OCT (6). Of note, 1 mg/mL OCT corresponds to 0.0001% OCT, which is drastically lower than concentrations in approved OCT-containing products (0.05 to 0.1%). Similar to E. coli (10), OCT inhibited growth of *E. hirae* in a concentration-dependent manner, increasing with elevating numbers of applied bacteria (Table 2). Hence, induction of cell death requires a higher number of OCT molecules per bacterial cell. Therefore, the concentration of OCT was always adjusted in each experiment to be below, at, and above the lethal concentration for the different bacterial load.

**TABLE 1:** Characteristic of tested *E. hirae* and B. subtilis strains including MIC values against OCT^*a*^

Strain	Relevant genotype	Defect	MIC (mg/L)	Source/reference
*E. hirae* ATCC 10541		WT	1	LGC Standards GmbH, Germany
B. subtilis 168	*trpC2*	WT	1	34
B. subtilis KS19	*lytABC*::*neo lytD*::*tet lytE*::*cat lytF*::*spc*	Multiple autolysins		39
B. subtilis bSS421	*amyE*::*spc PrpsD-sfgfp*			S. Syvertsson, unpublished
B. subtilis BS23^*b*^	*cat atpA-gfp Pxyl-'atpA*			40
B. subtilis ARK3	*clsA*(*ywnE*)::*tet clsB*(*ywjE*)::*spc ywiE*::*kan*	CL	0.5–1	41
B. subtilis KS119	*psd*::*MLS*	PE	0.5–1	41
B. subtilis AK0117B-A	*ugtP*::*MLS*	Glucolipid	0.5–1	A. Koh, unpublished
B. subtilis AK0118B-A	*mprF*::*kan*	Lysyl-PG	0.5–1	A. Koh, unpublished
B. subtilis AK066B	*pssA*::*spc*	PS	0.5–1	A. Koh, unpublished
B. subtilis AK066B	*yfnI*::*erm yqgS*::*spc ltaS*::*cat*	LTA	0.5	A. Koh, unpublished
B. subtilis AK094B	*tagO*::*MLS*	WTA	0.5	A. Koh, unpublished

a MIC was determined for ~1 × 10^6^ CFU/mL as a concentration of OCT where no visible growth was observed at 420 to 580 nm.

b Expression of the downstream part of the *atp* operon induced with 0.5% xylose.

**TABLE 2:** OCT induces growth inhibition and killing of *E. hirae*^*a*^

Concentration	OCT (%) determined by *E. hirae* CFU/mL:				
	1 × 10^6^	1 × 10^7^	1 × 10^8^	2.5 × 10^8^	1 × 10^9^
IC	0.0001	0.0001	0.0002	0.0004	0.0004–0.0008
LC	0.0002	0.0002	0.0004		0.0004

a Antimicrobial activity defined as an inhibitory concentration (IC) or lethal concentration (LC) was determined as a concentration where no visible growth was observed at 420 to 580 nm or no visible colonies were observed on diagnostic agar plates after previous exposure of OCT with *E. hirae* for 1 h. Data are expressed as medians of at least three independent experiments.

### OCT affects *E. hirae* cell envelopes.

**FIG 1:**
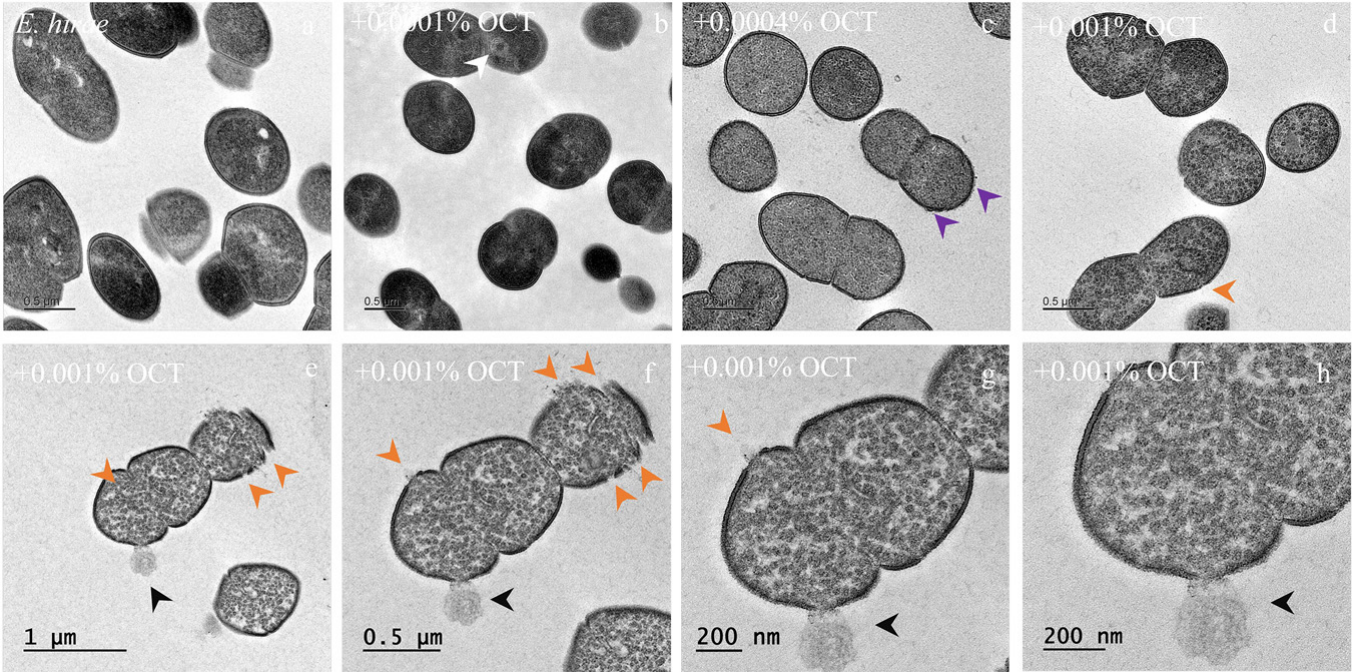
OCT induces structural changes in *E. hirae* cells. *E. hirae* samples of 2.5 × 10^8^ CFU/mL were incubated for 30 min in the absence (a) and presence of OCT below (0.0001% [b]), at (0.0004% [c]), and above lethal concentrations (0.001% [d]). Arrows indicate the following changes to the cell structure: damaged membrane surface (violet), ruptured membranes (orange) with some abnormal intracellular structures (white), and aggregation of the intracellular content (green). A representative picture after treatment with 0.001% OCT (e) was enlarged for better visualization of the membrane disruption and leakage of cytoplasmic content (black arrow) (g and h).

The cellular structure of *E. hirae* when treated with OCT was followed by electron microscopy. Cell wall and cytoplasmic membrane were intact and clearly resolved in the untreated bacteria (Fig. 1a). In contrast to E. coli (10), we could not observe massive changes on the cell wall when *E. hirae* was exposed to 0.0001% and 0.0004% OCT, below and at the lethal concentration, respectively. However, dramatic loss of the intracellular contrast showed some abnormal structures accumulated in the cytosol (Fig. 1b, white arrows) and some areas indicating damaged cell surface (Fig. 1c, violet arrows). Numerous granule-like structures (Fig. 1d to h) appeared in the cytoplasm when exposing the cells to 0.001% OCT (above the lethal concentration) as previously seen in E. coli (10). Crucially, perforation of the cell wall and the cytoplasmic membrane combined with leakage of cytoplasmic content was clearly visible at several locations of a single cell (Fig. 1e to h).

### OCT influences membrane integrity of *E. hirae*.

**FIG 2:**
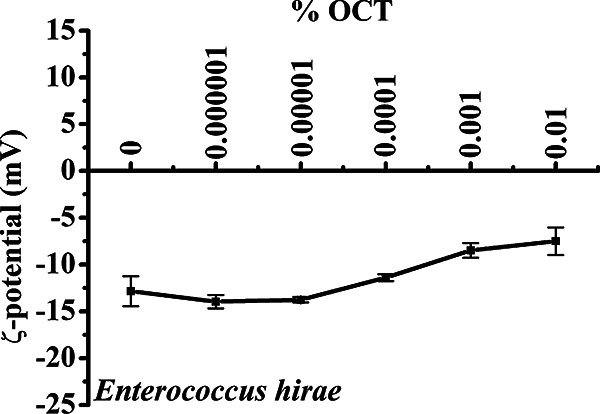
OCT neutralizes the surface charge of *E. hirae*. Neutralization of the surface charge of 1 × 10^7^ CFU/mL *E. hirae* in the absence and presence of 0.000001 to 0.01% OCT. Samples were incubated for 5 min before zeta potential measurement. Results were performed in duplicates of 30 independent calculations and analyzed at least three times.

Disturbances to the enterococcal cell wall and cytoplasmic membrane were clearly observed using electron microscopy. The question arose whether OCT also interacts with components of the cell wall in addition to cytoplasmic membrane. The cell wall of *E. hirae* is highly negatively charged due to the presence of (lipo)-teichoic acids, which might attract cationic OCT to the bacterial surface. Therefore, we first tested OCT’s capacity to neutralize the negative surface charge of the enterococcal membrane by measuring the zeta potential of *E. hirae* cells titrated with increasing concentrations of OCT (Fig. 2). *E. hirae* holds a negative zeta potential of −12.9 ± 1.5 mV, which started to increase around 0.0001% OCT, reaching a value of −7.5 ± 1.5 mV for 0.01% OCT. Hence, there is a strong correlation between induced changes in zeta potential and growth inhibitory activity against *E. hirae*. Although OCT cannot fully neutralize the enterococcal surface, a significant increase in zeta potential was observed at the killing concentration of 0.001%. This not only alludes to electrostatic interactions between positively charged OCT and negatively charged cell wall components (teichoic acids) but also strongly suggests that surface charge neutralization coincides with the key event of the OCT-mediated killing. As zeta potential did not become positive and under the assumption that interaction with bacterial surface is only electrostatic, we might assume that OCT penetrates directly into the cytoplasmic membrane and does not accumulate on the bacterial surface.

**FIG 3:**
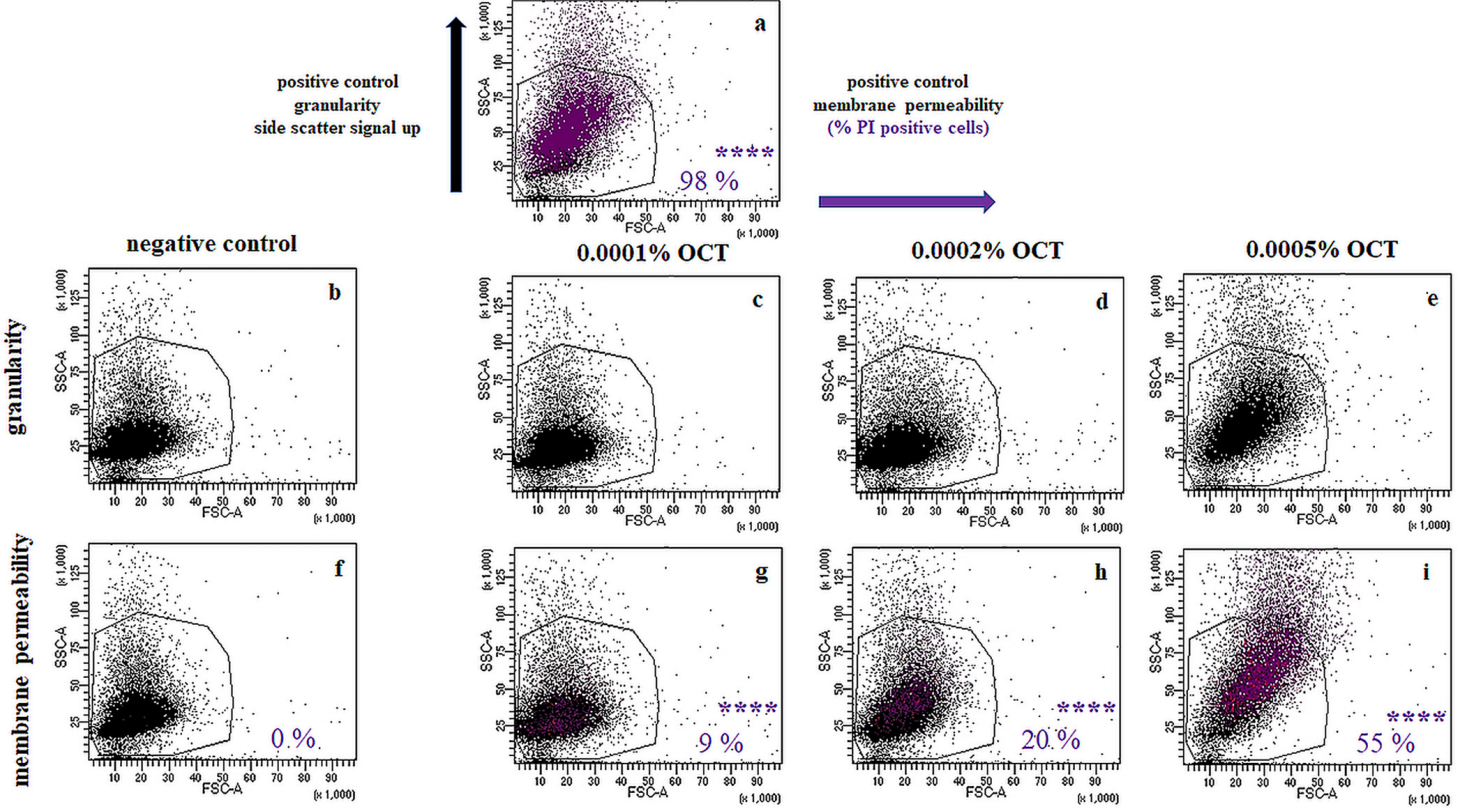
OCT affects membrane integrity and permeability of *E. hirae*. Side and forward scatter signals of flow cytometry experiments with *E. hirae* stained with PI in the presence of 0.8 μM SAAP-148 (synthetic antimicrobial and antibiofilm peptide) as a positive control for increased granularity and membrane permeability (a). Of note, PI fluorescence is observed upon binding to DNA to which it only gains access when membranes are compromised. As negative controls, the side and forward scatter signals of unstained (b) and stained with PI (f) *E. hirae* cells were recorded. The same experiments were repeated with OCT in the absence (c–e) and presence (g–i) of PI. Concentrations used are indicated in each graph. Here, only graphs for OCT concentrations below (0.0001%), at (0.0002%), and above the lethal concentration (0.0005%) are shown. Data are representative examples of a serial measurement from 0.0001% to 0.1% OCT obtained from at least two to three independent experiments. Membrane-active antimicrobial peptide SAAP-148 (33), our positive control, showed a rapid increase of 98% PI-positive cells concomitant with increased granularity. Significance for the increase in PI-positive cells were calculated using an unpaired two-sided *t* test.

To test whether OCT indeed targets the cytoplasmic membrane, we further assessed the membrane permeability using flow cytometry. For this aim, we used the large, normally membrane-impermeable, DNA intercalating fluorescent dye propidium iodide (PI). Indeed, incubation with OCT strongly increased intracellular PI staining in a concentration-dependent manner, reaching up to 55% of the cells within 20 min (Fig. 3). This indicates substantial damage to the cytoplasmic membrane with clear disruption of its barrier function. Of note, we use the terminology “increased permeability” to indicate abnormal, unspecific, and non-protein-facilitated passage of molecules across the cytoplasmic membrane. Furthermore, the flow cytometry confirmed an increase in granularity, showing a strong side scattering of *E. hirae* cells treated with OCT at or higher than 0.005% (above lethal concentration).

**FIG 4:**
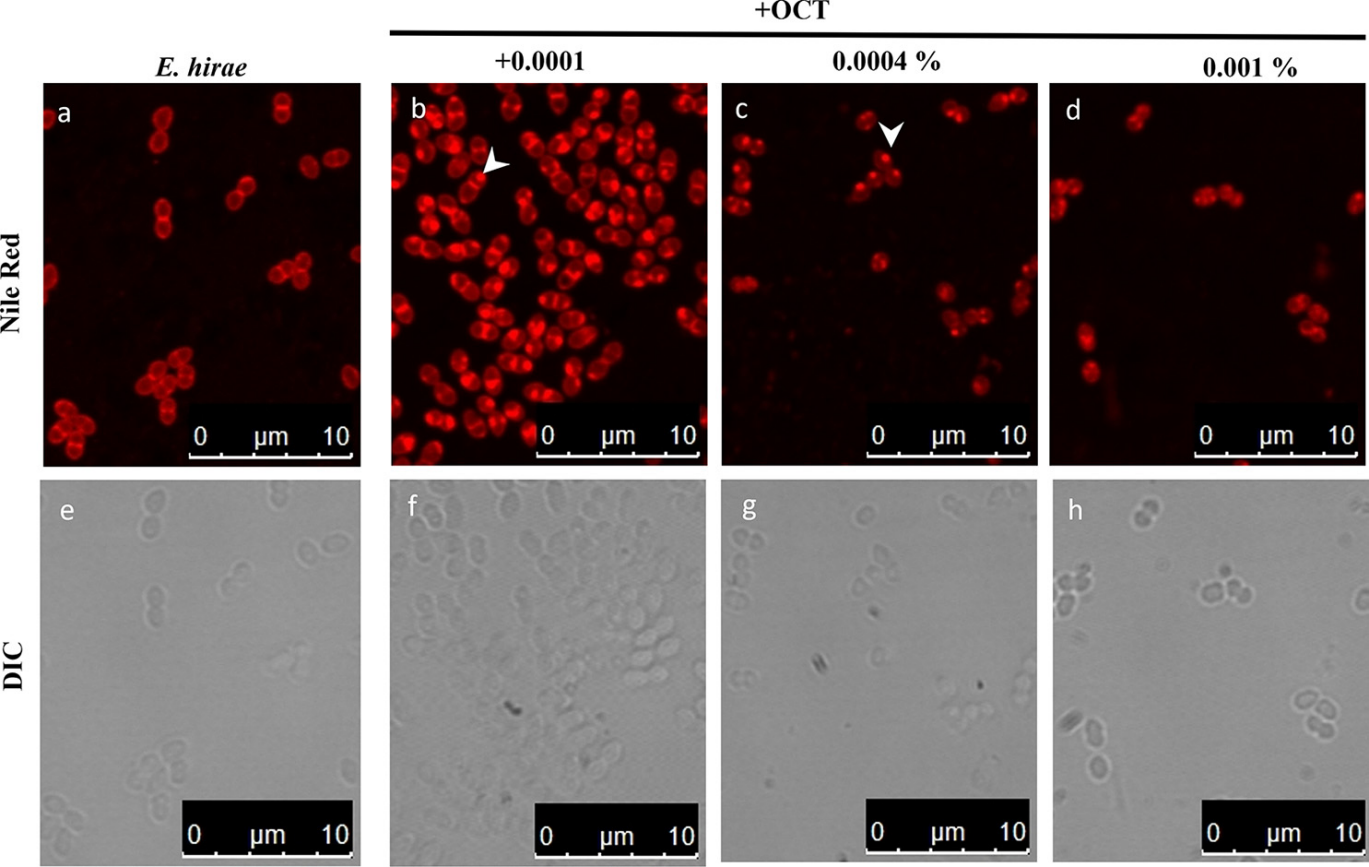
OCT affects membrane organization of *E. hirae* fluorescence and DIC images of *E. hirae* stained with the membrane dye Nile Red in the absence and presence of OCT at below (0.0001% [b]), at (0.0004% [c]), and above (0.001% [d]) lethal concentration of OCT. Nile Red staining of altered membrane areas is indicated by arrows. Nile Red is a membrane dye particularly sensitive to polarity of the environment and widely used to stain bacterial membranes. Images are representative examples of three independent experiments.

To confirm that the likely membrane invaginations observed using electron microscopy are not a consequence of the sample fixation process, and to confirm their membranous nature, we stained *E. hirae* with the membrane fluorescent dye, Nile Red. As shown in fluorescence microscopy images (Fig. 4), Nile Red stains *E. hirae* membranes in a homogenous manner, showing higher intensity in the septal region due to the presence of two adjacent membrane planes. At 0.0001% OCT, some Nile Red foci formed locally on one cell pole. With increasing OCT concentrations of 0.0004% (lethal), the staining was more heterogenous, and Nile Red foci became more distinct and remained only at one cell pole. With 0.001% OCT (above lethal concentration), the Nile Red signals were increasingly cytoplasmic, and the cells became smaller, indicating loss of turgor upon leakage of cytoplasmic content. Comparable to previous observations in E. coli (20), and in accordance with electron microscopy images, we assume that the presence of Nile Red foci indicates membrane areas with disturbed overall structure, potentially driven by OCT-induced local lipid disorder.

### Octenidine also influences membrane integrity of Bacillus subtilis.

**FIG 5:**
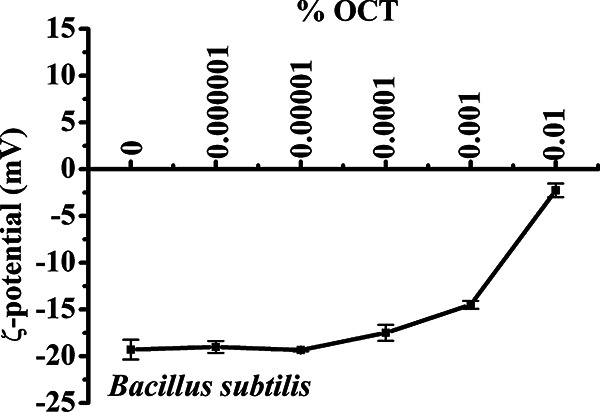
OCT neutralizes the surface charge of B. subtilis. Neutralization of the surface charge (zeta potential measurement) of 1 × 10^7^ CFU/mL B. subtilis in the absence and presence of 0.000001 to 0.01% OCT. Samples were incubated for 5 min before zeta potential measurement. Results were performed in duplicates of 30 independent calculations measured at least three times.

To confirm our findings with respect to OCT activity in another Gram-positive species and to further investigate the nature of the Nile Red foci observed in *E. hirae* (Fig. 4) as well as in E. coli (10), we used B. subtilis. Besides differences in cell morphology and membrane composition, more comprehensive studies with B. subtilis are feasible due to the availability of strains bearing mutations in production of membrane lipids relevant for our experiments. First, we assessed B. subtilis susceptibility to OCT, which turned out to be similar to that of *E. hirae*. The concentration of 0.0004% OCT was inhibitory to 1 × 10^8^ CFU/mL, which corresponds to an inhibitory concentration (IC) of 4 mg/L (data not shown). For 1 × 10^7^ CFU/mL, the IC was calculated with maximum 1 mg/L OCT (Table 1). Second, to provide evidence that OCT also neutralizes the surface charge of B. subtilis, we performed zeta potential measurements. In accordance with *E. hirae*, B. subtilis surface charge of approximately −20 mV started to increase around the killing concentration of 0.0001% (Fig. 5). Treatment with above lethal concentrations resulted in complete neutralization of the surface charge, highlighting again the importance of electrostatic forces for interaction of OCT with cell components exposed on the bacterial surface.

**FIG 6:**
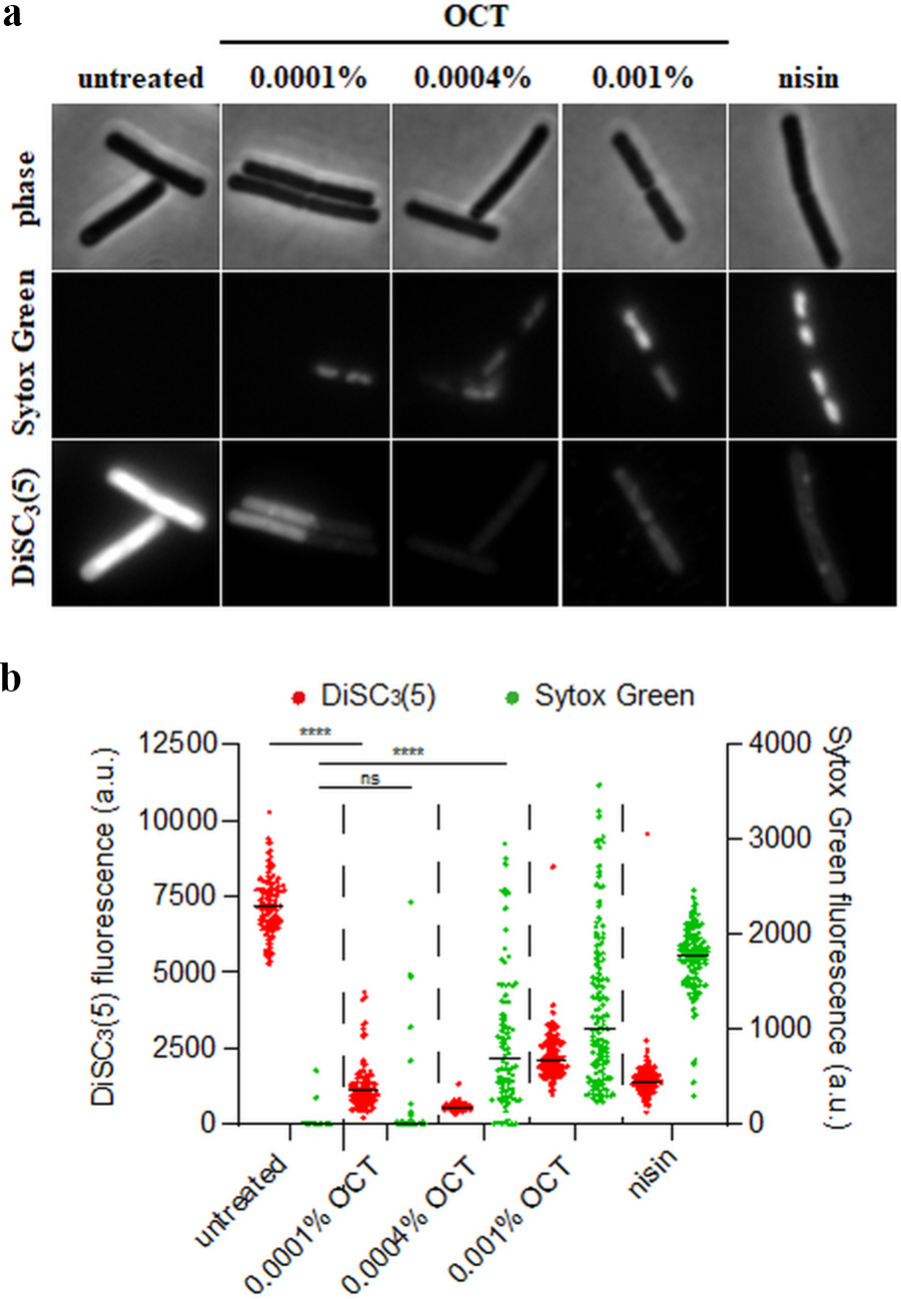
OCT disrupts the membrane barrier function in B. subtilis. (a) Phase contrast and fluorescence microscopy images of B. subtilis 168 (wild type) cells costained with the voltage-sensitive dye DiSC_3_(5) and the membrane permeability indicator Sytox Green and incubated in the absence and presence of different concentrations of OCT (5 min). As a positive control, the transmembrane potential and the membrane barrier functions were disrupted by the pore forming lantibiotic nisin (10 μM). DiSC_3_(5) is a voltage-sensitive fluorescent dye which, due to its cationic and hydrophobic nature, can accumulate in polarized cells and is released upon depolarization (28, 29). Sytox Green, conversely, is a large, membrane-impermeable fluorescent dye, which is DNA-intercalating and fluoresces when large pores are formed in the cell membrane (26, 27). (b) Quantification of DiSC_3_(5) and Sytox Green fluorescence for individual cells from the data set shown in panel A (*n* = 90 to 130 cells). Median fluorescence intensity is indicated with a black line, together with *P* values of an unpaired, two-sided *t* tests. ****, *P* < 0.0001; n.s., nonsignificant difference.

We then performed combined Sytox Green and DiSC_3_(5) fluorescence microscopy to simultaneously investigate the effect of OCT on membrane permeability (26, 27) and membrane potential (28, 29). At the lowest concentration tested, OCT caused a heterogeneous depolarization of B. subtilis, as demonstrated by a reduction in DiSC_3_(5) fluorescence (Fig. 6). With increasing OCT concentration, the depolarization became more homogenous and extensive. At higher concentrations, OCT also permeabilized the cytoplasmic membrane as indicated by staining with Sytox Green (Fig. 6).

**FIG 7:**
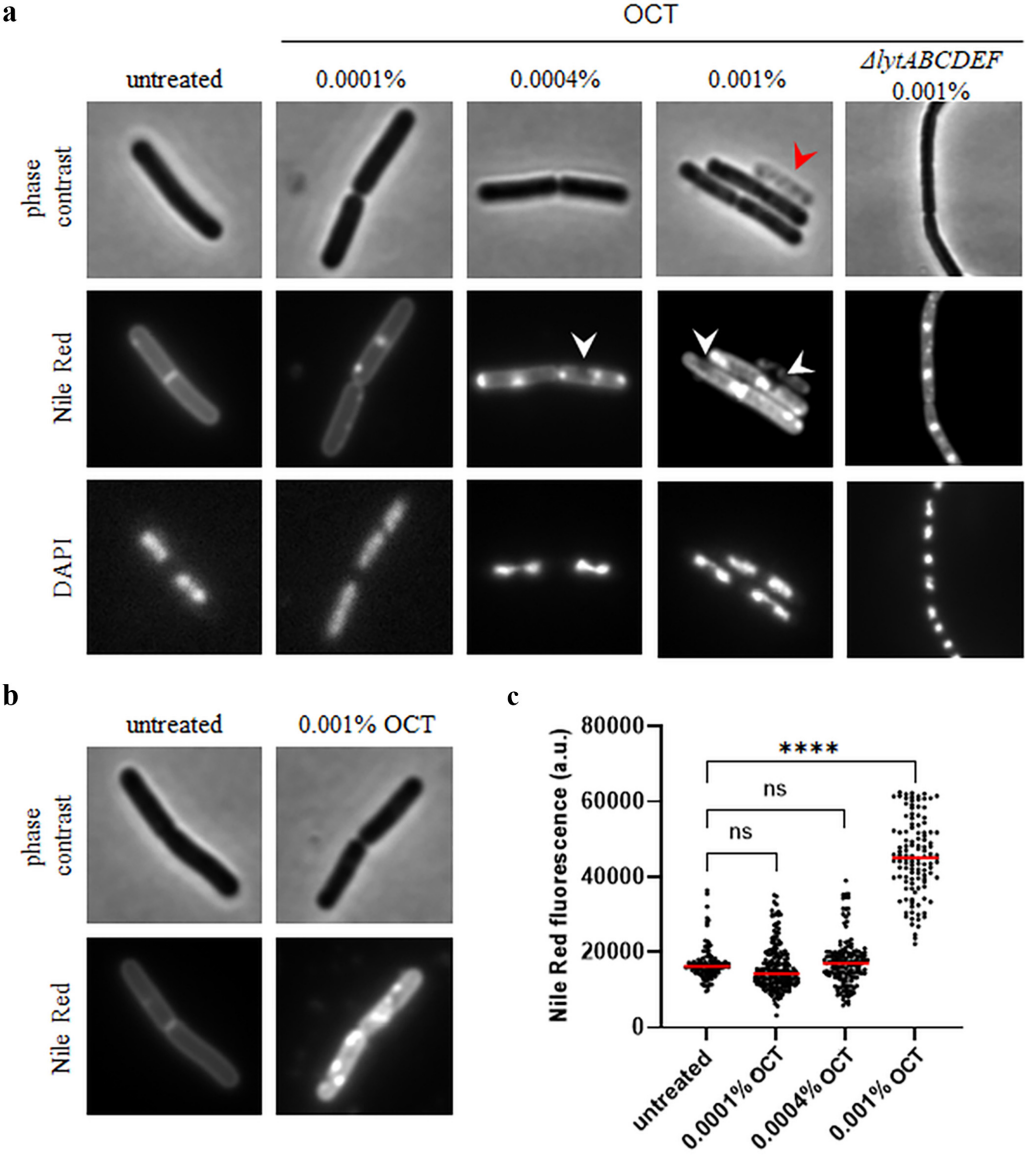
OCT disturbs membrane organization and fluidity in B. subtilis. (a) Phase contrast and fluorescence microscopy images of B. subtilis 168 (wild type) cells costained with the membrane dye Nile Red and the DNA dye DAPI in the absence and presence of different concentrations of OCT (5 min). Membrane areas weakly stained by Nile Red and fully lysed cells are highlighted with white and red arrows, respectively. For comparison, cells lacking several autolytic enzymes (Δ*lytABCDEF*), which do not undergo octenidine-induced cell lysis, are included. To highlight local differences in staining, the contrast values are not preserved between the individual fluorescence image fields. (b) Phase contrast and fluorescence microscopy images of B. subtilis stained with Nile Red in the absence and presence of 0.001% OCT (5 min). Here, the fluorescence images retain identical contrast settings allowing intensity comparison. (c) Quantification of Nile Red fluorescence for individual cells from the same imaging data set shown in panels a and b (*n* = 118 to 199 cells). Median cell Nile Red fluorescence intensities are indicated with red lines, together with *P* values of an unpaired, two-sided *t* tests. ****, *P* < 0.0001; n.s., nonsignificant difference. See Fig. S1 in the supplemental material for a larger field of view with more cells.

Next, we performed combined Nile Red and 4′,6-diamidino-2-phenylindole (DAPI) fluorescence microscopy (Fig. 7a; see also Fig. S1 in the supplemental material). As shown in Fig. 7a, Nile Red stains untreated B. subtilis membranes in a homogeneous manner without a visible preference for certain membrane areas. Upon incubation with OCT, however, we observed the emergence of brightly stained Nile Red foci, which developed into larger membrane areas at the highest OCT concentration. These membrane irregularities were qualitatively very similar to those observed in *E. hirae*. Interestingly, the overall Nile Red staining levels were strongly increased in cells treated with OCT (Fig. 7b), and when quantified, fluorescence in 0.001% OCT-treated cells was significantly higher compared to untreated ones (Fig. 7c). It has been previously shown that Nile Red fluorescence intensity is influenced by changes in fluidity of the membrane (30, 31); therefore, it is likely that this increase in Nile Red fluorescence is caused by a strong increase in overall membrane fluidity.

We also observed membrane areas with very low Nile Red staining and, at the highest concentrations, fully lysed cells (highlighted by white and red arrows, respectively) (Fig. 7a). To investigate whether the weakly stained membrane areas were due to OCT-induced membrane disturbances or linked to the developing cell lysis process, we repeated the Nile Red fluorescence microscopy in a B. subtilis strain deficient of its major autolysins (Δ*lytABCDEF*). As expected, no lysis was observed microscopically when the cells were treated with a lethal dose of OCT for 30 min (see Fig. S2 in the supplemental material). Whilst incubation of Δ*lytABCDEF* cells with OCT still induced a spotty Nile Red membrane stain, the weakly stained membrane areas were absent in this strain, indicating that this phenomenon is not a direct consequence of OCT. Rather, the emergence of such areas is a secondary phenomenon associated with the cell lysis process.

**FIG 8:**
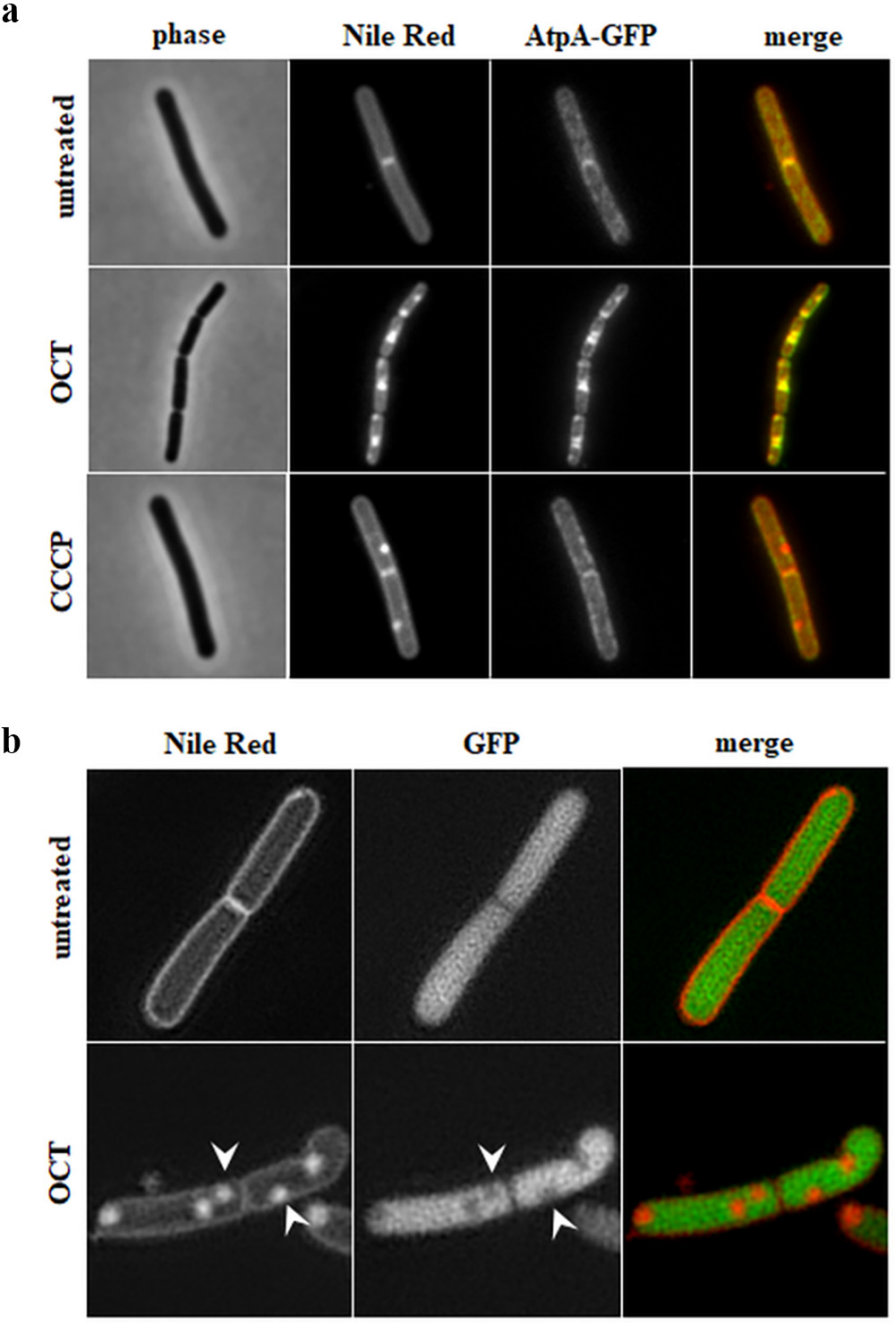
OCT-induced bright Nile Red foci are caused by membrane invaginations in B. subtilis. (a) Phase contrast and fluorescence microscopy images of Nile Red-stained B. subtilis BS23 (AtpA-GFP) cells expressing AtpA-GFP in the absence and presence of 0.001% OCT (5 min). Note the colocalization of induced Nile Red and AtpA-GFP foci, which indicates membrane invaginations as the cause for high local Nile Red intensity. As a control, cells were incubated with 100 μM of the protophore CCCP (5 min), which induces invagination-independent Nile Red foci associated with high local membrane fluidity that do not influence AtpA-GFP localization pattern. (b) Structural illumination microscopy (SIM) of B. subtilis cells expressing sfGFP from a strong ribosomal promoter (PrpsD) and stained with Nile Red in the absence and presence of OCT (0.001%, 5 min). Note that the OCT-induced membrane invaginations exclude soluble, cytoplasmic GFP (white arrows). Strains used include the following: B. subtilis BS23 (AtpA-GFP) and bSS421 (PrpsD-sfGFP).

To confirm that the strongly fluorescent Nile Red foci are indeed membrane invaginations, we followed the colocalization between the Nile Red signal and the FoF1 ATP synthase, which under normal conditions exhibits a uniform localization pattern along the membrane (31). As shown in Fig. 8a, incubation with OCT induced strong clustering of the FoF1 ATP synthase, which clearly colocalized with the fluorescent Nile Red foci. Hence, consistent with an invagination model, the foci are indeed also enriched in cytoplasmic membrane proteins. Conversely, this is not observed for the control CCCP (carbonyl cyanide 3-chlorophenylhydrazone), which induces areas of high local membrane fluidity that are also preferentially stained by Nile Red but are not associated with membrane invaginations (31). To confirm these findings through a more direct approach, we performed dual-color structured illumination microscopy (SIM) of Nile Red-stained B. subtilis cells that constitutively express high levels of cytoplasmic green fluorescent protein (GFP). In untreated cells, we observed a uniform staining of the membrane, and the cytoplasm was flooded with GFP (Fig. 8b). However, upon treatment with OCT, the Nile Red foci were clearly associated with local exclusion of the GFP signal (white arrows). This provides strong, direct evidence that OCT indeed induces membrane invaginations. Whilst likely, the presence of local membrane folds unfortunately renders it impossible to judge whether the brightly stained Nile Red membrane areas are also altered in local fluidity and disorder.

### Action of OCT is not phospholipid specific but favors hydrophobic over electrostatic forces.

Given that bacteria are relatively diverse in their phospholipid composition which, in turn, may affect OCT activity, we investigated OCT susceptibility to a set of B. subtilis strains lacking specific phospholipid species. In addition, we also tested the relevance of wall- and lipoteichoic acids in this context. As shown in Table 1, the MIC values of OCT did not change in mutants lacking any of the potential specific targets known to be present in B. subtilis membranes (glucolipids, teichoic acids, PE, PS, CL, and lysyl-PG), thus clearly pointing toward a non-lipid-specific mode of action of OCT.

**FIG 9:**
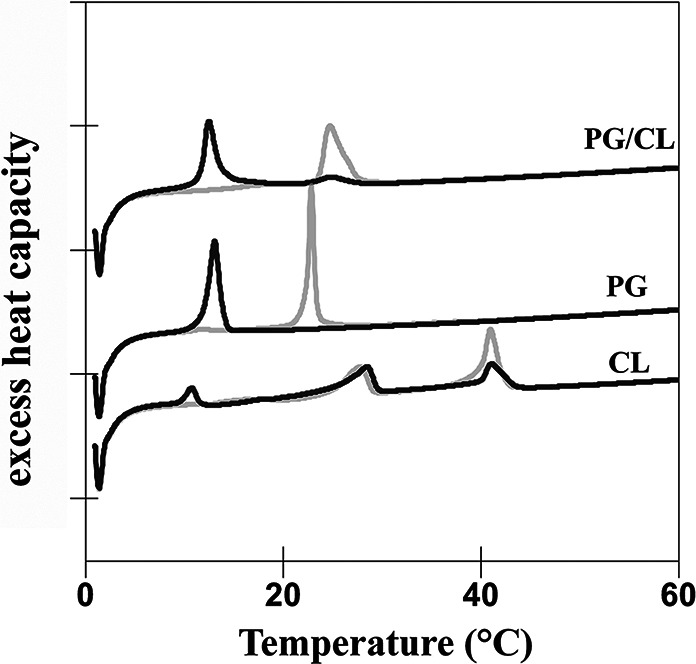
OCT changes membrane properties of PG/CL model membranes. Thermodynamic studies of PG/CL mixture, PG, and CL in the absence (gray line) and presence of OCT (black lines). Heating scans of PG/CL model membranes as observed at a molar lipid-to-OCT ratio of 6:1. Scan rate was 30°C/h. Data are representative examples of at least two independent experiments. Thermotropic parameter of PG (42) and CL (43) correspond to published thermotropic behavior of those lipids.

We reported earlier about significant defects in the acyl chain region upon OCT’s action on bilayer membranes composed of the most abundant PLs, a mixture of PE/PG (10). The question arose whether OCT’s activity on lipid bilayers is determined by CL. Due to its ability to induce membrane curvature (17), CL has the potential to significantly change the membrane properties of *E. hirae* (PG/CL) and B. subtilis (PE/PG/CL). To test this, CL was introduced into the various membrane formulations, and their thermotropic behavior upon OCT treatment was examined by differential scanning calorimetry. The results showed, independent of phospholipid composition used, the same thermotropic profile as previously described: the main phase transition of all membranes disappeared with increasing OCT concentration, accompanied by a new phase transition at much lower melting temperatures (10). However, we noticed a slight preference for zwitterionic over negatively charged PLs, as the main phase transition was abolished at different lipid-to-OCT molar ratios in the following order, PE > PG > CL (10) (Fig. 9), indicating a very narrow range of OCT specificity toward phospholipids. Introduction of CL into PG (PG/CL) (Fig. 9; Table 3) or PE/PG (PE/PG/CL) (see Fig. S3 in the supplemental material and reference 10) membranes did not change respective profiles.

**TABLE 3:** OCT affects thermotropic parameters of model membranes^*a*^

Membrane characterization	Low-temp transition (SGII/LR1 → Lβ')		Pretransition (Lβ' → Pβ')		Main transition (Lβ/Pβ' → Lα)	
	ΔH (kcal/mol)	*T*_MAX_ (°C)	ΔH (kcal/mol)	*T*_MAX_ (°C)	ΔH (kcal/mol)	*T*_MAX_ (°C)
PG/CL					9.9	24.4
+ OCT					1.5	25.0
					9.5	12.5
PG			1.01	11.6	7.9	22.9
+ OCT					9.6	13.1
CL	1.8	10.6	12.3	28.5	10.3	40.9
+ OCT	2.5	10.8	9.63	28.6	9.4	41.1

a Phase transition temperature (*T*_m_) and corresponding enthalpies (ΔH_cal_) of model membranes composed of PG/CL mimicking *E. hirae* cytoplasmic membrane in the absence and presence of OCT at 6:1 lipid-to-OCT molar ratios. Data are means of two independent experiments with deviation less than 1%.

Similar observations were made when we performed leakage experiments with different membrane compositions and properties (Table 4). Although significant differences in the degree of leakage were observed at lower OCT concentrations, higher concentrations induced complete leakage for all formulations.

**TABLE 4:** OCT permeabilizes model membranes to a similar extent^*a*^

Model membranes	Anionic content (~%)	% Leakage	
		(L: OCT = 8:1)	(L: OCT = 4:1)
PG	100	50	100
PG/CL	100	10	90
PE	0	73	100
PE/PG	20	30	90
PE/CL	10	90	100
PE/PG/CL	30	25	96
E. coli polar lipid extract	30	90	100

a Leakage of ANTS from ANTS/DPX-loaded model membranes with composition of 50 μM PG, PG/CL, PE/CL, PE/CL, PE/PG/CL, or E. coli lipid extracts titrated with OCT concentrations corresponding to lipid-to-OCT molar ratios ranging from 62.5:1 to 2:1. Data are means of two or more independent experiments performed in duplicates. 100% leakage is related to maximal leakage induced by triton. Leakage was significantly induced at 0.0004% OCT, which corresponds approximately to L: OCT of 8:1 and was complete at 0.0008% OCT. Higher concentration did not reach higher leakage.

**FIG 10:**
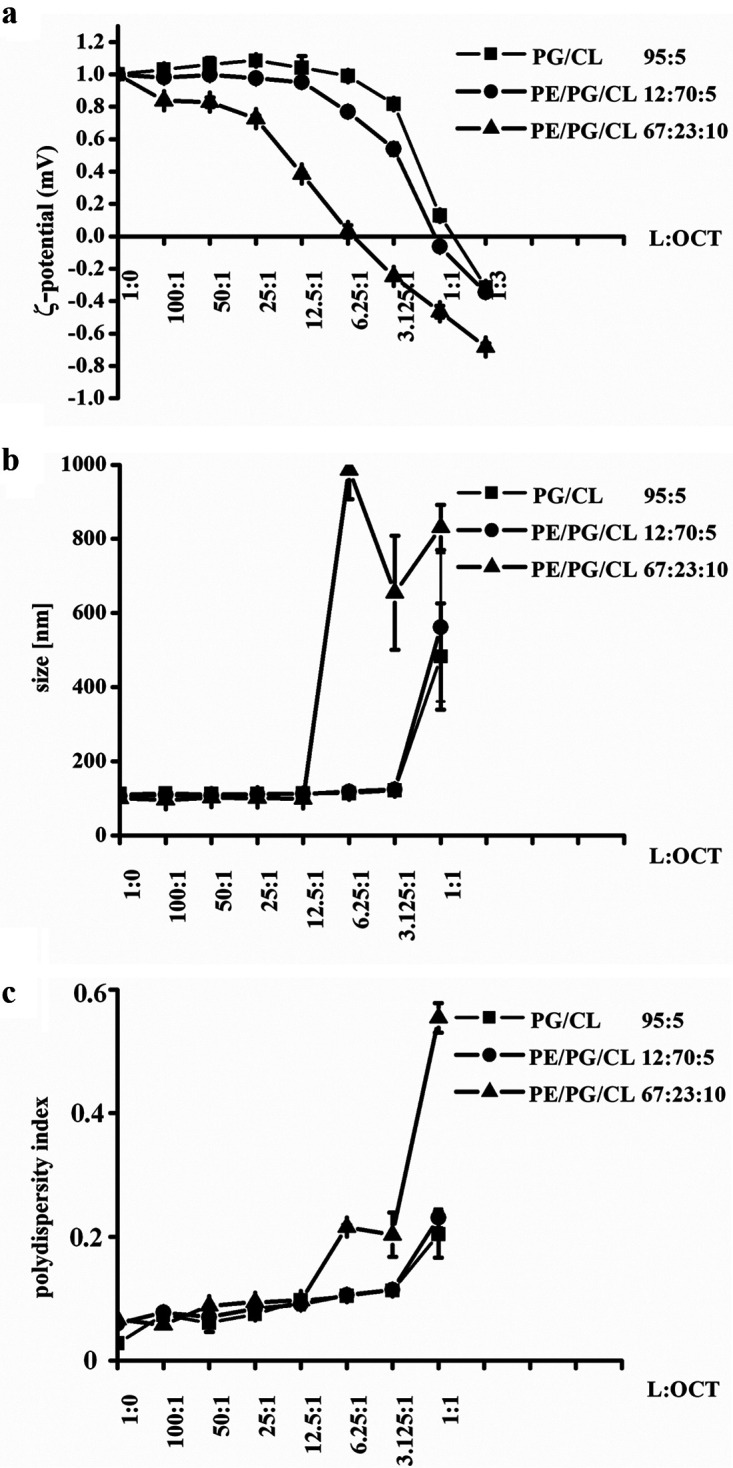
OCT preferentially neutralizes surface charge of the zwitterionic cytoplasmic membrane models. Measurement of zeta potential (a), size (b), and polydispersity index (c) of 50 μM PG/CL (squares), PE/PG/CL (circle, highly anionic), and PE/PG/CL (triangle, less anionic) in the absence and presence of 0.5 to 50 μM OCT. Samples were incubated for 5 min before analysis. Results were performed in duplicates of 30 independent calculations measured at least two times. Of note, the size and polydispersity index of the vesicles did not change before neutralization, and then a significant increase was observed for all systems, which indicated a collapse of the system due to flocculation of the samples. Indeed, floccules were already visible to the eye.

To test if OCT interaction with PLs is electrostatic, we performed zeta potential measurements (Fig. 10) with model membranes of different anionic content resembling those found in cytoplasmic membranes of *E. hirae* (PG/CL, 100% anionic), B. subtilis (PE/PG/CL, 85% anionic), and E. coli (PE/PG/CL, 33% anionic). OCT neutralized surface charge of all membrane models; however, neutralization of highly anionic *E. hirae* and B. subtilis model membranes started above leakage concentrations. Similar results were observed by Rzycki et al. (32). This suggest that hydrophobic forces dominate during membrane disruption. The zeta potential recorded for E. coli model membranes is primarily due to interaction of OCT with PE, as OCT induced the same profile on vesicles composed of solely PE (see Fig. S4 in the supplemental material). Although we did not expect such a profile for zwitterionic surfaces, we assume that, in contrast to PG, PE’s capability for hydrogen bonding with OCT-reactive groups may provoke different organization of OCT on the surface of PE. Nevertheless, our data suggest that hydrophobic interactions are maximized upon OCT interaction with phospholipids. In summary, our *in vivo* and *in vitro* data strongly support lipid headgroup-independent targeting of bacterial membranes as the primary mechanism underlying the broad and potent activity.

### Conclusion.

In summary and based on the biophysical and cellular data presented herein, the mode of action of OCT for Gram-positive bacteria can be described as follows. Upon adherence to bacteria, OCT begins to neutralize bacterial surface charge due to electrostatic interactions and immediately penetrates through the thick cell wall of Gram-positive bacteria to reach the cytoplasmic membrane. The hydrocarbon chains of OCT rapidly interfere with the fatty acyl chains of the cytoplasmic membrane, thereby inducing a strong disorder due to a hydrophobic mismatch. On a cellular level, OCT-induced changes to the cytoplasmic membrane are sufficient to depolarize by increasing ion conductivity and even induce membrane leakage of larger molecules. Furthermore, OCT induces extensive disturbance to the membrane structure as evidenced by large membrane invaginations and rupture. On a molecular level, OCT lowers the phase transitions of bacterial phospholipids leading to formation of membrane structures with packing disorder, likely explaining the increased permeability. The described mode of action explains OCT’s lack of selectivity toward microorganisms with varying cell envelope structures and compositions, thus explaining its strong and broad antiseptic activity. Crucially, such a rapid bactericidal mechanism targeting critical and essential membrane properties renders bacterial resistance development toward OCT less likely.

## MATERIALS AND METHODS

### Chemicals.

Octenidine (OCT) dissolved in pure water was provided by Schülke & Mayr GmbH (Austria) and SAAP-148 (synthetic antimicrobial and antibiofilm peptide) by Jan Wouter Drijfhout (LUMC, Leiden, The Netherlands) with the purity of >95% as analyzed by high-pressure liquid chromatography (HPLC) (33). DAPI, DiSC_3_(5), Nile Red, CCCP, nisin, and L-dopamine were purchased from Sigma-Aldrich. Propidium iodide (PI) and Sytox Green were purchased from Thermo Fisher Scientific. The following phospholipids (>99% purity) were ordered from Avanti Polar Lipids (USA): E. coli polar lipid extracts, DPPG (1,2-dipalmitoyl-sn-glycero-3-[phospho-rac-(1-glycerol)]), DPPE (1,2-dipalmitoyl-sn-glycero-3-phosphoethanolamine), DMPG (1,2-dimyristoyl-sn-glycero-3-[phospho-rac-(1-glycerol)]), DMPE (1,2-miristoyl-sn-glycero-3-phosphoethanolamine), TMCL (1,1’,2,2’-tetramyristoyl cardiolipin), POPG (1-palmitoyl-2-oleoyl-sn-glycero-3-[phospho-rac-(1-glycerol)]), and POPE (1-palmitoyl-2-oleoyl-sn-glycero-3-phosphoethanolamine). ANTS (8-aminonaphthalene-1,3,6-trisulfonic acid, disodium salt) and DPX (*p*-xylene-bis-pyridinium bromide) were obtained from Molecular Probes (USA).

### Microorganisms and culture.

The FDA-approved *E. hirae* strain for antimicrobial susceptibility testing (ATCC 10541; LGC Standards GmbH, Germany) was cultivated in brain heart infusion broth (BHIB) (Carl Roth, Germany). B. subtilis wild-type 168 (34) and its derivative strains were grown in lysogeny broth (LB) (Roth, Austria, or Oxoid, UK). All strains used in this study are listed in Table 1. If not otherwise stated, overnight cultures were prepared from single colonies by incubating the cells at 37°C and shaking at 200 rpm in BHIB or LB. The main culture was made with an inoculum of 0.05 optical density at 600 nm (OD_600_). The cultures were grown until the mid-logarithmic phase, which was reached after 3.5 to 4 h.

### Antimicrobial activity.

Antimicrobial activity was assessed as a function of growth. Briefly, *E. hirae*/B. subtilis in the mid-logarithmic phase was washed once with sodium phosphate-buffered saline (PBS) (20 mM Na_2_HPO_4_/NaH_2_PO_4_, 130 mM NaCl, pH 7.4) and inoculated at 1 × 10^6^ CFU/mL, 1 × 10^7^ CFU/mL, 1 × 10^8^ CFU/mL, and 1 × 10^9^ CFU/mL, respectively, in fresh BHIB or LB in the presence and absence of OCT (final concentrations up to 8 mg/L). Growth curves were recorded automatically for 24 h at 37°C under shaking (300 rpm) by Bioscreen C (Oy Growth Curves Ab Ltd, Finland). MIC for bacterial concentration of ~1 × 10^6^ CFU/mL was estimated as the lowest concentration of OCT in milligrams per liter where no visible bacterial growth was observed. Inhibitory concentration (IC) for higher bacterial loads follows the same description and was estimated in the same concentration range but expressed as corresponding %OCT (1 to 8 mg/L corresponds to 0.0001 to 0.0008% OCT), which allows direct comparison of the data to clinically used concentrations of OCT. Of note, MIC/IC for B. subtilis was estimated only for 1 × 10^6^ CFU/mL and 1 × 10^7^ CFU/mL bacterial loads. For elucidation of bactericidal concentration, different concentrations of bacteria were exposed to OCT for 1 h in PBS at 37°C before they were plated on BHIB agar plates. Lethal concentrations (LC) were estimate as a concentration where no visible colonies were observed after 1 day incubation of the plates at 37°C.

### Experimental section for *E. hirae*.

A set of experiments were performed in analogy to our previous work (10), and a detailed description is provided in the supplemental material. Briefly, electron and fluorescence microscopy were carried out with the same workflow and setup that we used for E. coli and published before (10, 35). The same is true for zeta potential measurements (10, 36) and flow cytometry.

### Experimental section for B. subtilis. (i) Fluorescence microscopy (B. subtilis).

Overnight cultures were grown at 30°C in LB supplemented with 0.2% glucose. B. subtilis Δ*lytABCDEF* overnight cultures were grown in the presence of 5 μg/mL chloramphenicol. Cells were diluted 100-fold in LB and grown at 37°C until an OD_600_ of 0.3. Samples were then incubated with different OCT concentrations (0.0001, 0.004, or 0.001%) and stained simultaneously with either 200 nM Sytox Green and 1 μM DiSC_3_(5) or with 0.125 μg/mL Nile Red and 0.5 μg/mL DAPI (4′,6-diamidino-phenylindole) for 5 min at 37°C upon shaking. A total of 0.5 μL of each sample was immobilized on microscope slides covered with a thin layer of H_2_O/1.2% agarose and imaged immediately. Microscopy was performed using a Nikon Eclipse Ti equipped with Nikon Plan Apo 100×/1.40 Oil Ph3 objective, CoolLED pE-300 light source, and Photometrics Prime sCMOS camera. Images were captured with MetaMorph 7.7 (Molecular Devices) and analyzed with ImageJ/Fiji (37).

### (ii) Structured illumination microscopy (B. subtilis).

Cells were prepared and immobilized on 1.2% agarose slides as described above. To reduce binding of Nile Red to the coverslip surface, which can suppress the structured illumination pattern, the coverslips were coated with l-dopamine as described before (28). In brief, l-dopamine (2 mg/mL freshly dissolved in 1 mM Tris pH 8.0) was added to the coverslip and incubated at room temperature for 30 min. The excess l-dopamine and Tris were then removed by aspiration and submersion in H_2_O followed by evaporation at 37°C for 30 min. Dual-color two-dimensional (2D)-SIM was performed using Nikon N-SIM equipped with 488- and 561-nm lasers, Nikon CFI SR HP Apochromat TIRF 100×/1.49 oil objective, and Andor iXon DU-897 camera. Image capture and SIM reconstruction was carried out with NIS Elements 5.21 (Nikon).

### Experimental section for biophysical studies.

Preparation of model membranes (10, 38), vesicle leakage assay (10, 38), and calorimetric measurements using a Microcal VP-DSC high-sensitivity differential scanning calorimeter (Microcal, USA) or Nano DSC (TA Instruments, Germany) (10, 38) were also performed in analogy to our previous work (10). A detailed description of the model membranes and methods is provided in the supplemental material.

### Zeta potential and vesicle size measurements.

According to methods published previously (10, 36), the same procedure using a Zetasizer NANO (Malvern Instruments, Germany) was adapted for measurements with 50 μM liposomes composed of indicated molar binary/ternary phospholipid mixtures DMPG/TMCL (80:20 mol), POPG/TOCL (95:5 mol), POPE/POPG/TOCL (12:70:5 mol), and POPE/POPG/TOCL (67:23:10 mol). OCT was used at ~0.00003 to 0.003% corresponding to 0.5 to 50 μM to adjust lipid-to-OCT molar ratios to 100:1 to 3:1. Within the same instrument and setup, the size and polydispersity index of the vesicles were determined.

### Data statistical analysis.

Statical analysis was assessed using an unpaired two-sided *t* test (Microsoft Excel). Unless otherwise stated, error bars represent standard deviation (SD) from three independent biological replicates. Significance for *t* test was assumed as follows: ****, *P* < 0.0001; ***, *P* < 0.001; **, *P* < 0.01; *, *P* < 0.05; n.s., not significant.
