# Supplementary material for: Disruption of the Cytoplasmic Membrane Structure and Barrier Function Underlies the Potent Antiseptic Activity of Octenidine in Gram-Positive Bacteria
Source: Appl Environ Microbiol. 2022 Apr 28;88(10):e00180-22. doi: 10.1128/aem.00180-22 (PMC9128513; doi:10.1128/aem.00180-22)
Supplement: Supplemental file 1 — Supplemental methods and Fig. S1 to S4. Download aem.00180-22-s0001.pdf, PDF file, 0.4 MB [file aem.00180-22-s0001.pdf]

Disruption of the cytoplasmic membrane structure and barrier function underlines the potent antiseptic activity of octenidine in Gram-positive bacteria

Nermina Malanovic[1#], Jessica A. Buttress[3], Djenana Vejzovic[1], Ayse Ön[1], Paulina Piller[1], Dagmar Kolb[2,4], Karl Lohner[1,2] and Henrik Strahl[3]

[1]Institute of Molecular Biosciences, Biophysics Division, University of Graz, NAWI Graz, Graz, Austria

[2]Bio TechMed Graz, 8010 Graz, Austria

[3]Centre for Bacterial Cell Biology, Biosciences Institute, Faculty of Medical Sciences, Newcastle University, Baddiley-Clark Building, Richardson Road, Newcastle upon Tyne, NE2 4AX, United Kingdom

[4]Core Facility Ultrastructure Analysis, Center for Medical Research, Medical University of Graz, Stiftingtalstrasse 24, 8010 Graz, Austria

* djenana.vejzovic@hotmail.com

§ ayse_oen@hotmail.com

#Corresponding author:

email: nermina.malanovic@uni-graz.at

Institute of Molecular Biosciences, Biophysics Division, University of Graz, Humboldtstrasse 50/III, 8010 Graz, Austria.

Running title: Antimicrobial mode of action of octenidine

Supplementary Methods

A set of experiments were performed in analogy to our previous work [1].

Electron microscopy

Electron microscopy was carried out with the same workflow and set up we used for *E. coli* and published before [1]. Samples were prepared with $2.5x10^8$ CFU/ml *E. hirae* from the mid-logarithmic culture and incubated for 30 min at 37°C in presence of below (0.0001 %), at (0.0004 %) and above the lethal concentration (0.001 %) of OCT. Experiments were performed twice and images were taken of at least six cuts of which representative once are shown.

Zeta potential and vesicle size measurements

Mid-logarithmic *E. hirae*/*B. subtilis* cultures were diluted to $1x10^7$ CFU/ml in HEPES buffer (10 mM HEPES, 140 mM NaCl, pH 7.4) and incubated with OCT from $1x10^{-9}$ to $1x10^{-3}$% (5 min, room temperature) before measuring the samples in a Zetasizer NANO (Malvern Instruments, Germany) according to methods published previously [1,2].

Flow cytometry

600-1200 µl of the $1x10^6$ CFU/ml cell suspension were incubated for 5 min at room temperature with 1 µg/ml propidium iodide (PI) in the dark. PI fluorescence was measured with BD LSR Fortessa™ in real-time using the BD FACS Diva Software. Signals from side and forward scatter were also collected, as they relate to complexity and cell size of bacteria. After 30 sec, OCT in several dilutions from 0.00005 to 0.001 % was added to the labelled bacterial cells, mixed and fluorescence of PI was followed for 5-20 min. We used the membrane-active antimicrobial peptide SAAP-148 [3] as a positive control, which is known to permeabilise

bacterial membranes. FACS experiments in the presence of OCT were repeated in the absence of PI.

Fluorescence microscopy (*E. hirae*)

Assays for *E. hirae* were performed as described previously [1,4]. A mid-logarithmic growth-phase culture of *E. hirae* was diluted in PBS to 2.5x10^8 CFU/ml. 200 µl of this cell suspension was incubated with OCT concentrations below (0.0001 %), at (0.0004 %) and above (0.001 %) the lethal concentration (20 min; 37°C; 300 rpm) before they were stained with 10 µg/ml of the fluorescent membrane dye Nile Red. The used microscope and set-up was according to Wolinski *et al.* [5]. Nile Red was excited at 561 nm and fluorescence emission was detected between 570-750 nm.

Vesicle leakage assay

For leakage experiments, large unilamellar vesicles composed of POPE, POPG, POPE/TOCL (80:10 mol), POPG/TOCL (80:20 mol), POPE/POPG/TMCL (6:2:1 mol) or *E. coli* polar lipid extract (PE 67 %, PG 23.2 % and CL 9.8 %) were prepared in 10 mM HEPES buffer pH 7.4 containing 68 mM NaCl, 12.5 mM ANTS and 45 mM DPX. Leakage of the aqueous content of the 50 µM ANTS/DPX loaded lipid vesicles upon incubation with OCT-to-lipid molar ratios from 1:25 to 1:1.5 was determined as described previously [1]. Fluorescence emission was recorded at 37°C as a function of time before and after the addition of OCT. Triton was used as a positive control for maximum induced leakage of fluorescent dye.

Differential Scanning Calorimetry (DSC)

Calorimetric measurements were performed using a Microcal VP-DSC high-sensitivity differential scanning calorimeter (Microcal, USA) or Nano DSC (TA Instruments, Germany)

as described before [6]. DSC measurements were carried out in presence and absence of OCT on model membranes composed of major phospholipids (DMPG, TMCL and DMPG/TMCL mixture (80:20 molar) of the Gram-positive membranes [7]. Total lipid concentration was 1 mg/ml and the used OCT concentration was calculated to correspond to a lipid-to-OCT molar ratio of 6:1. Preparation of the model membranes was done as described before [6].

Supplementary Figures

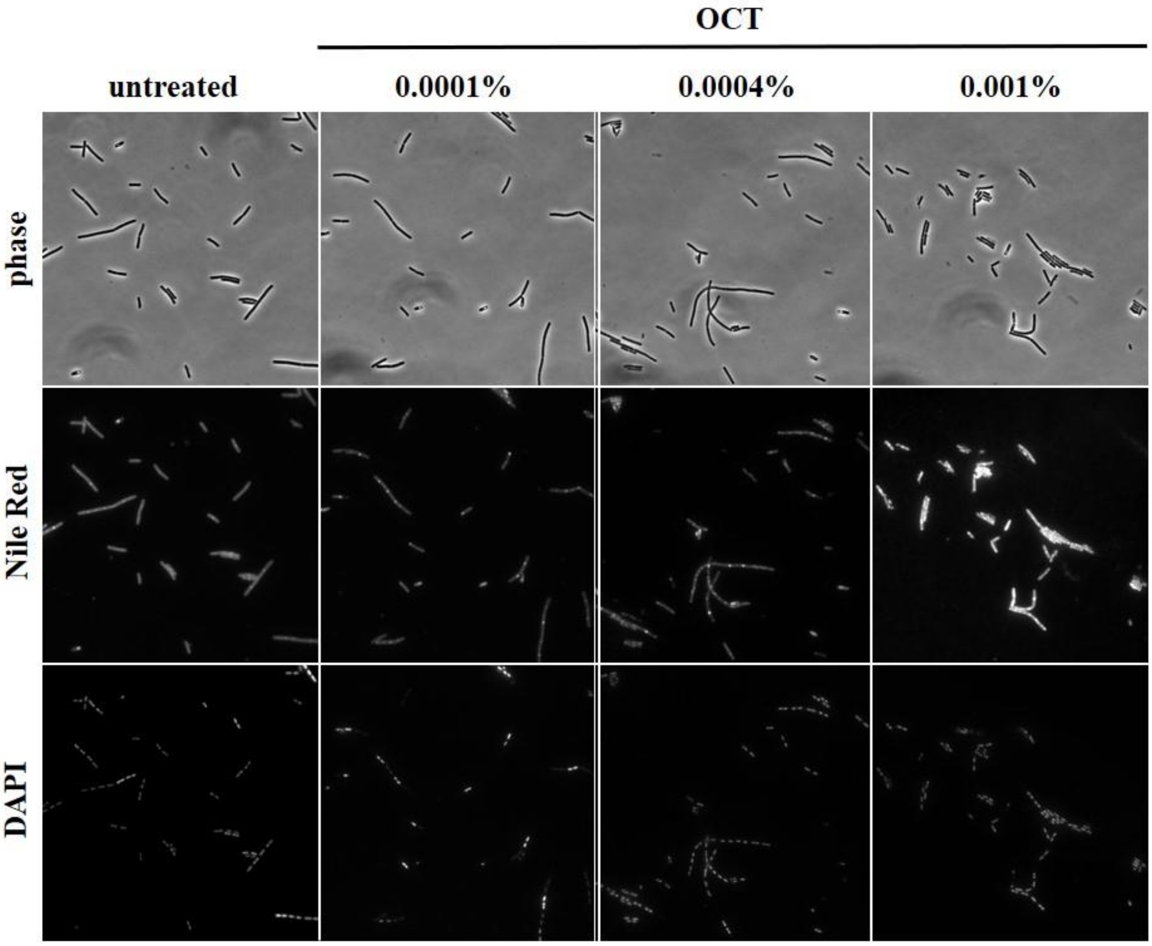

Supplementary Figure S1: OCT disturbs membrane organisation and fluidity in *B. subtilis;* a larger field of view.

Phase contrast and fluorescent microscopy images of *B. subtilis* cells co-stained with the membrane dye Nile Red and the DNA dye DAPI, in the absence and presence of different concentrations of OCT (5 min). These images show a larger field of view with more cells of the data show in Figure 7. Strain used: *B. subtilis 168* (wild type).

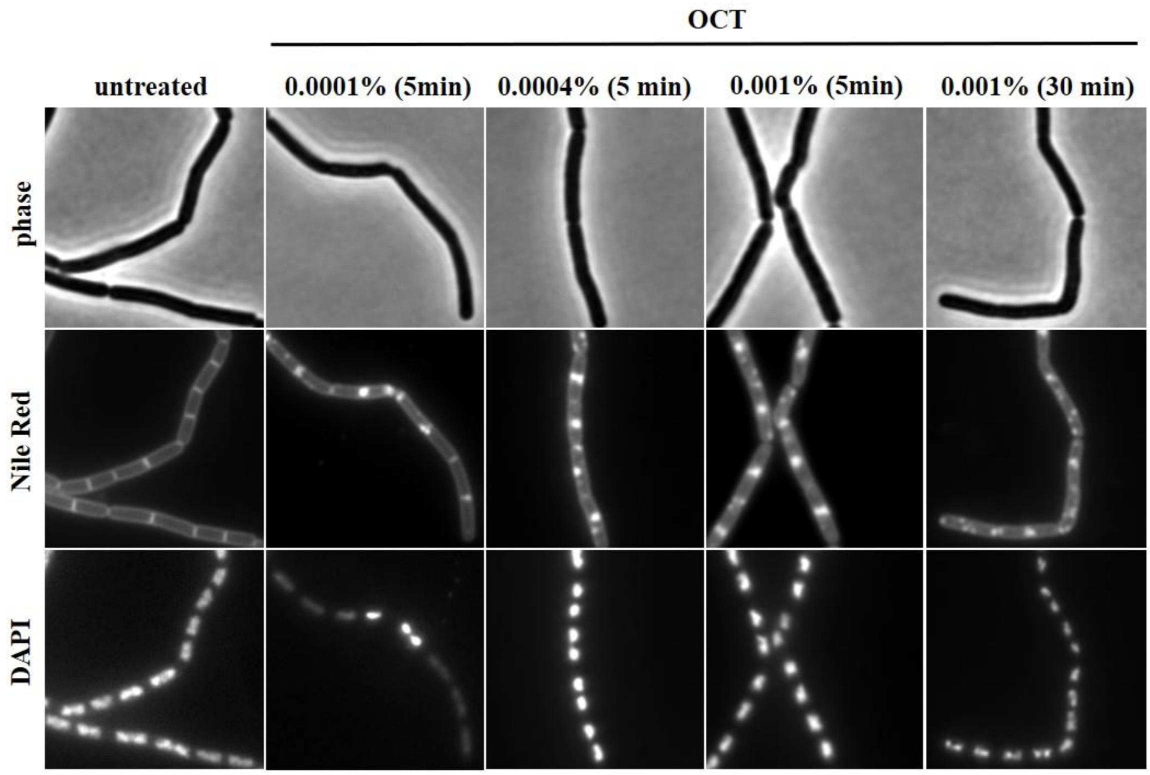

Supplementary Figure S2: Weakly Nile Red-stained stained membrane areas are a manifestation of cell lysis.

Phase contrast and fluorescent microscopy images of *B. subtilis* cells deficient for cell wall autolytic enzymes LytC-F, co-stained with the membrane dye Nile Red and the DNA dye DAPI, and incubated in the absence and presence of different concentrations of OCT (5-30 min). Note the lack of weakly stained membrane areas observed in lysing wild type cells whilst the brightly stained foci associated with membrane invaginations are still present. Strain used: *B. subtilis* KS19 (Δ*lytABCDEF*). Due to the lack of cell wall hydrolase activity, these cells exhibit a chainy cell morphology as they cannot complete septal cleavage.

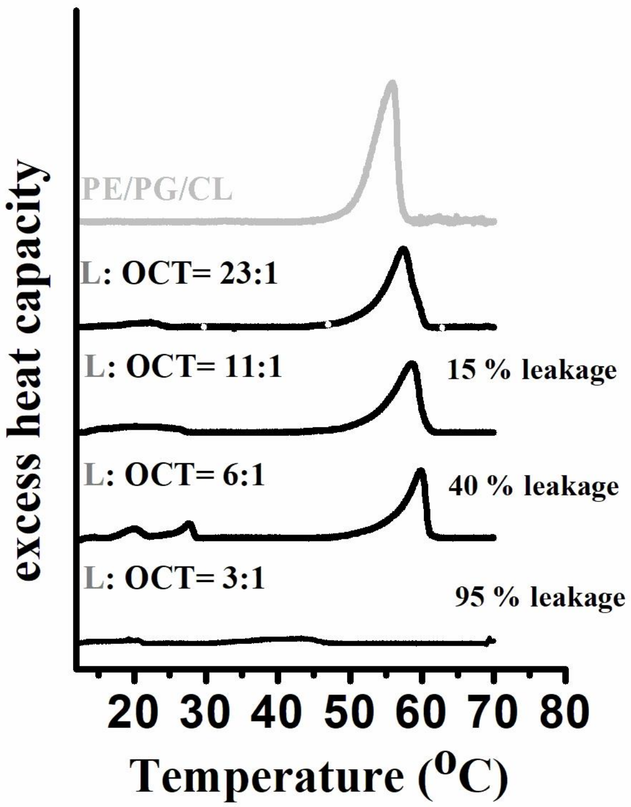

Supplementary Figure S3. OCT affects membrane properties of PE/PG/CL membrane models as determined by calorimetric measurements. Thermodynamic studies of PE/PG/CL mixture (grey line) in the presence of indicated OCT concentration (black lines). Heating scans of PE/ PG/CL model membranes as observed at the indicated molar lipid-to-OCT ratios. Scan rate was 30°C/h. Data are representative examples of at least two independent experiments

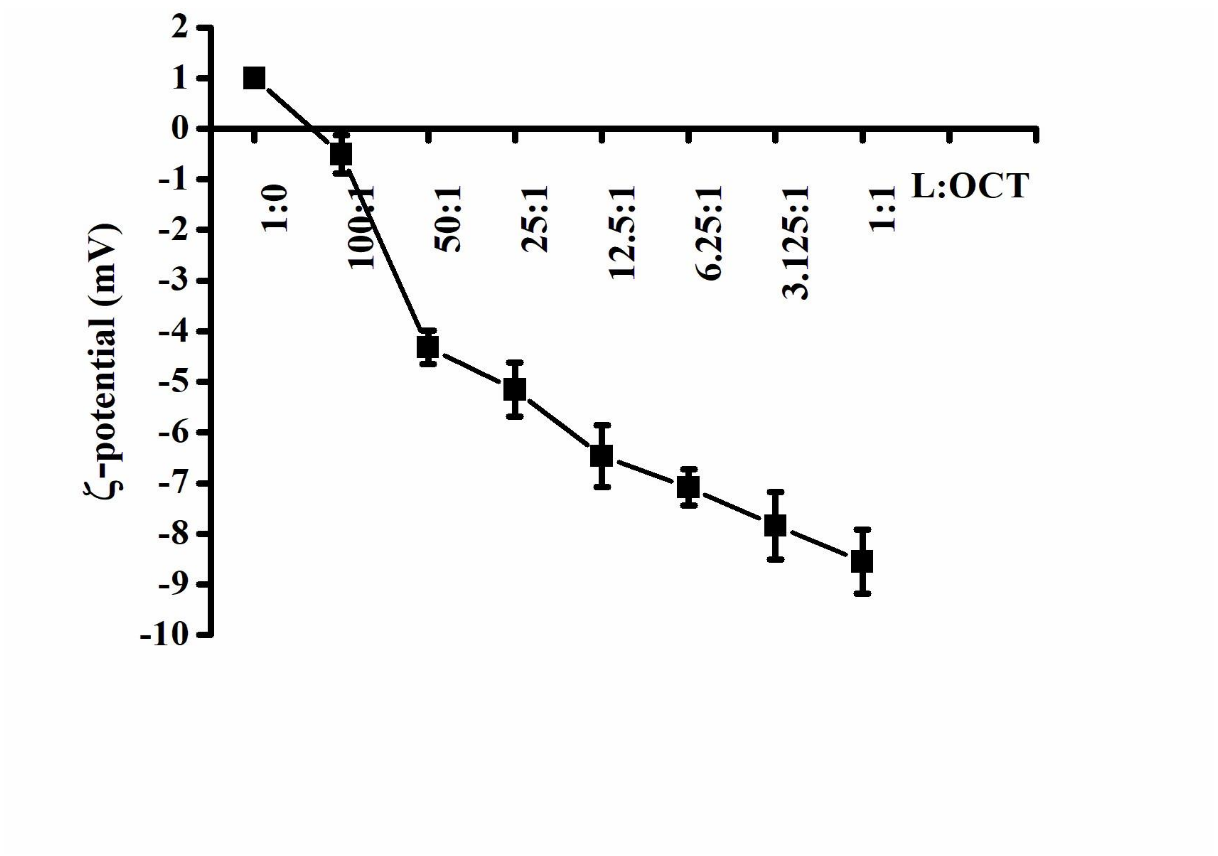

Supplementary Figure S4. OCT neutralizes surface charge of the PE membranes. Measurement of zeta potential of 50 µM PE in absence and presence of 0.5-50 µM OCT. Samples were incubated for 5 min before analysis. Results were performed in duplicates of 30 independent calculations measured at least two times.

Supplementary References

[1] Malanovic N, Ön A, Pabst G, Zellner A, Lohner K. Octenidine: Novel insights into the detailed killing mechanism of Gram-negative bacteria at a cellular and molecular level. Int J Antimicrob Agents 2020;56:106146.

[2] Pérez-Peinado C, Dias SA, Domingues MM, Benfield AH, Freire JM, Rádis-Baptista G, Gaspar D, Castanho MARB, Craik DJ, Henriques ST, Veiga AS, Andreu D. Mechanisms of bacterial membrane permeabilization by crotalicidin (Ctn) and its fragment Ctn(15-34), antimicrobial peptides from rattlesnake venom, J Biol Chem 2018;293;1536-1549.

[3] de Breij A, Riool M, Cordfunke RA, Malanovic N, de Boer L, Koning RI, Ravensbergen E, Franken M, van der Heijde T, Boekema BK, Kwakman PHS, Kamp N, El Ghalbzouri A, Lohner K, Zaat SAJ, Drijfhout JW, Nibbering PH. The antimicrobial peptide SAAP-148 combats drug-resistant bacteria and biofilms, Sci Tranls Med 2018;10(423):eaan4044

[4] Scheinpflug K, Krylova O, Nikolenko H, Thurm C, Dathe M. Evidence for a novel mechanism of antimicrobial action of a cyclic R-,W-rich hexapeptide, PLoS One 2015;e0125056.

[5] Wolinski H, Kohlwein SD. Microscopic and spectroscopic techniques to investigate lipid droplet formation and turnover in yeast, Methods Mol Biol (Clifton, N.J.) 2015;1270:289-305.

[6] Malanovic N, Leber R, Schmuck M, Kriechbaum M, Cordfunke RA, Drijfhout JW, de Breij A, Nibbering PH, Kolb D, Lohner K. Phospholipid-driven differences determine the action of the synthetic antimicrobial peptide OP-145 on Gram-positive bacterial and mammalian membrane model systems, Biochim Biophys Acta 2015;1848;2437-2447.

[7] Malanovic N, Lohner K. Gram-positive bacterial cell envelopes. The impact on the activity of antimicrobial peptides. Biochim Biophys Acta 2016;1858:936-946.
